# Supplementary material for: Impact of continuous labor companion- who is the best: A systematic review and meta-analysis of randomized controlled trials
Source: PLoS One. 2024 Jul 23;19(7):e0298852. doi: 10.1371/journal.pone.0298852 (PMC11265680; doi:10.1371/journal.pone.0298852)
Supplement: S1 Table — (DOCX) [file pone.0298852.s001.docx]

S1 Table. Effectiveness of trained vs. untrained labour companion.

| Outcome | | No. of Participants (Studies) | RR (95% CI) | P value | Heterogeneity  (I^2^) | Test for subgroup difference (p) |
| --- | --- | --- | --- | --- | --- | --- |
| 1. Spontaneous vaginal delivery | Trained | 12634  (13 RCTs) | 1.07(1.03,1.11) | 0.0005 | 0.42 | 0.34 |
|  | Untrained | 1177  (6 RCTs) | 1.16(0.99,1.35) | 0.07 | 0.90 |  |
| 2. Duration of labour | Trained | 4598  (14 RCTs) | 0.31(0.17,0.44) | 0.0001 | 0.76 | 0.99 |
|  | Untrained | 824  (3 RCTs) | 0.31(0.16,0.46) | 0.0001 | 0.72 |  |
| 3. Cesarean section | Trained | 13468  (18 RCTs) | 1.22(1.05,1.42) | 0.01 | 0.44 | 0.02 |
|  | Untrained | 1612  (6 RCTs) | 2.16(1.37,3.40) | 0.0009 | 0.54 |  |
| 4. Instrumental delivery | Trained | 12978  (16 RCTs) | 1.13(1.02,1.24) | 0.01 | 0.31 | 0.75 |
|  | Untrained | 977  (5 RCTs) | 1.20(0.83,1.74) | 0.34 | 0.14 |  |
| 5. Oxytocin for labour induction | Trained | 11296  (15 RCTs) | 1.12(1.00,1.25) | 0.04 | 0.73 | 0.52 |
|  | Untrained | 1662  (6 RCTs) | 1.52(0.60,3.82) | 0.38 | 0.97 |  |
| 6. Analgesic usage | Trained | 10419  (14 RCTs) | 1.05(1.00,1.10) | 0.04 | 0.55 | 0.39 |
|  | Untrained | 1300  (4 RCTs) | 1.15(0.94,1.42) | 0.17 | 0.36 |  |
| 7. Tocophobia | Trained | 10148  (8 RCTs) | 1.34(1.14,1.57) | 0.0005 | 0.59 | 0.004 |
|  | Untrained | 985  (3 RCTs) | 1.84(1.60,2.12) | 0.0001 | 0 |  |
| 8. 5 min APGAR < 7 | Trained | 11883  (11 RCTs) | 1.45(0.99,2.13) | 0.06 | 0.05 | 0.94 |
|  | Untrained | 656  (5 RCTs) | 1.51(0.55,4.20) | 0.43 | 0.47 |  |
