# Supplementary material for: Impact of continuous labor companion- who is the best: A systematic review and meta-analysis of randomized controlled trials
Source: PLoS One. 2024 Jul 23;19(7):e0298852. doi: 10.1371/journal.pone.0298852 (PMC11265680; doi:10.1371/journal.pone.0298852)
Supplement: S2 Table — (DOCX) [file pone.0298852.s002.docx]

S2 Table. Effectiveness of a labour companion before and after 2000.

| Outcome | | No. of Participants (Studies) | RR (95% CI) | P value | Heterogeneity  (I^2^) | Test for subgroup difference (p) |
| --- | --- | --- | --- | --- | --- | --- |
| 1. Spontaneous vaginal delivery | Studies before 2000 | 4642  (11 RCTs) | 1.10(1.05,1.14) | 0.0001 | 0.27 | 0.63 |
|  | Studies after 2000 | 9169  (8 RCTs) | 1.07(1.01,1.15) | 0.03 | 0.71 |  |
| 2. Duration of labour | Studies before 2000 | 3824  (10 RCTs) | 0.16(0.06,0.26) | 0.001 | 0.44 | 0.004 |
|  | Studies after 2000 | 1598  (7 RCTs) | 0.53(0.30,0.77) | 0.0007 | 0.74 |  |
| 3. Cesarean section | Studies before 2000 | 4741  (12 RCTs) | 1.27(1.01,1.58) | 0.04 | 0.38 | 0.19 |
|  | Studies after 2000 | 10339  (12 RCTs) | 1.62(1.21,2.16) | 0.001 | 0.76 |  |
| 4. Instrumental delivery | Studies before 2000 | 4843  (13 RCTs) | 1.19(1.00,1.40) | 0.05 | 0.42 | 0.30 |
|  | Studies after 2000 | 9112  (8 RCTs) | 1.07(0.99,1.17) | 0.10 | 0 |  |
| 5. Oxytocin for labour induction | Studies before 2000 | 4033  (11 RCTs) | 1.15(0.99,1.34) | 0.07 | 0.71 | 0.80 |
|  | Studies after 2000 | 8925  (10 RCTs) | 1.12(0.98,1.28) | 0.09 | 0.81 |  |
| 6. Analgesic usage | Studies before 2000 | 3856  (11 RCTs) | 1.08(0.99,1.18) | 0.07 | 0.64 | 0.79 |
|  | Studies after 2000 | 8863  (7 RCTs) | 1.06(1.00,1.14) | 0.06 | 0.45 |  |
| 7. Tocophobia | Studies before 2000 | 3281  (6 RCTs) | 1.43(1.23,1.67) | 0.0001 | 0.22 | 0.75 |
|  | Studies after 2000 | 7852  (5 RCTs) | 1.50(1.17,1.93) | 0.001 | 0.79 |  |
| 8. 5 min APGAR < 7 | Studies before 2000 | 2873  (6 RCTs) | 1.61(0.96,2.69) | 0.07 | 0 | 0.81 |
|  | Studies after 2000 | 9666  (10 RCTs) | 1.78(0.92,3.47) | 0.09 | 0.37 |  |
