# Supplementary material for: Impact of continuous labor companion- who is the best: A systematic review and meta-analysis of randomized controlled trials
Source: PLoS One. 2024 Jul 23;19(7):e0298852. doi: 10.1371/journal.pone.0298852 (PMC11265680; doi:10.1371/journal.pone.0298852)

**Instrumental vaginal delivery**

**
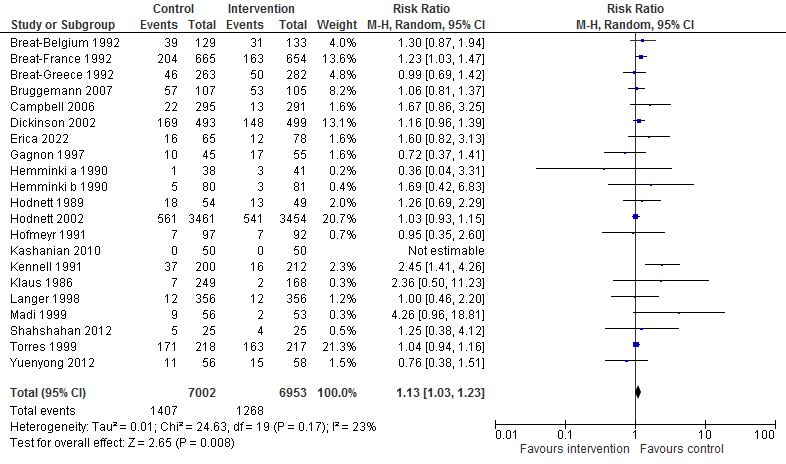
**

**Funnel Plot**

**
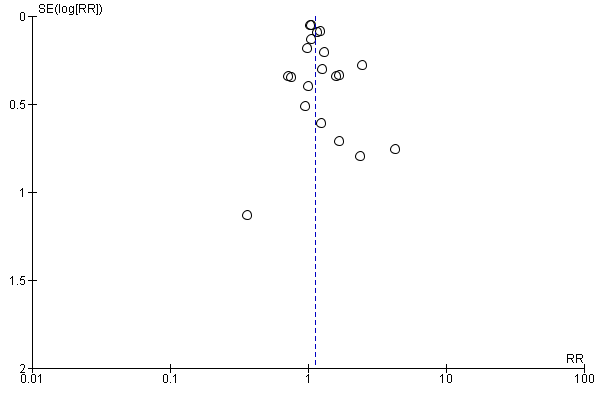
**

**Timeline**

**
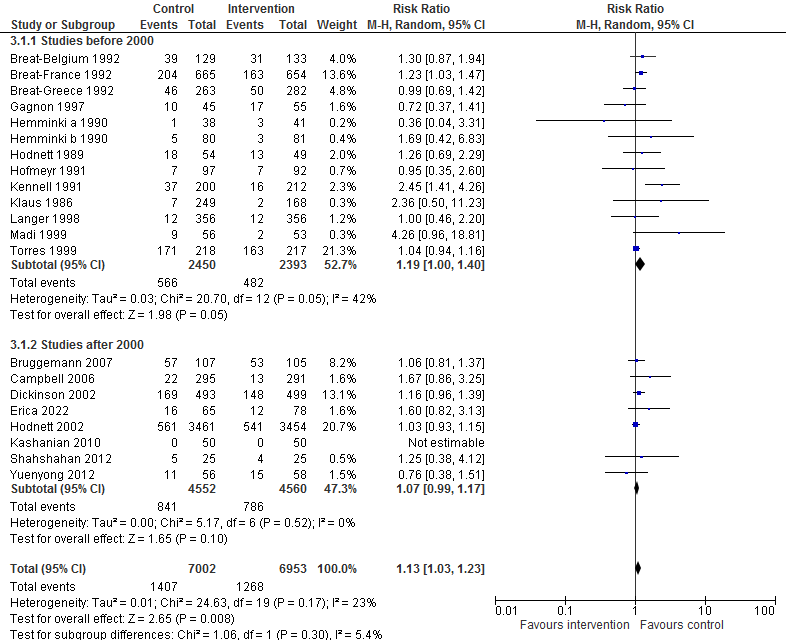
**

**Trained vs Untrained Companion**

**
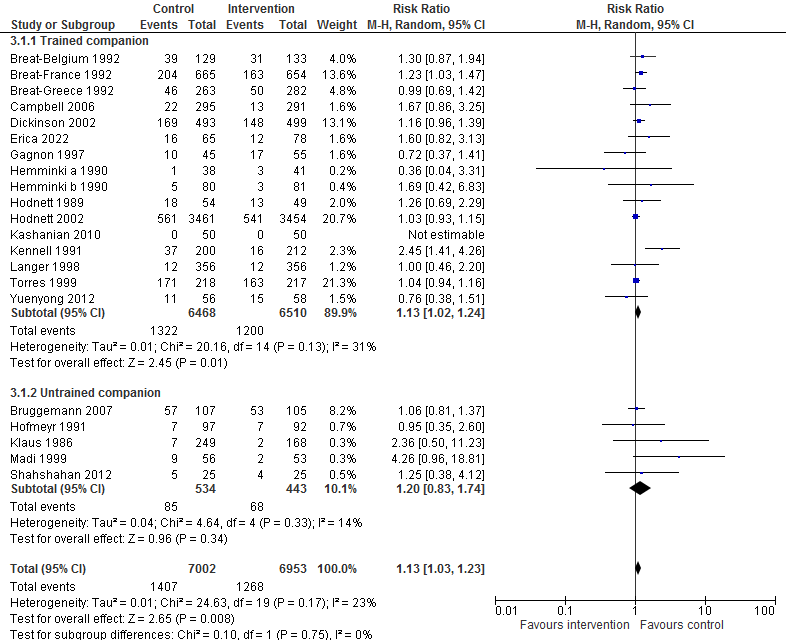
**

**Familiar vs Unfamiliar Companion**

**
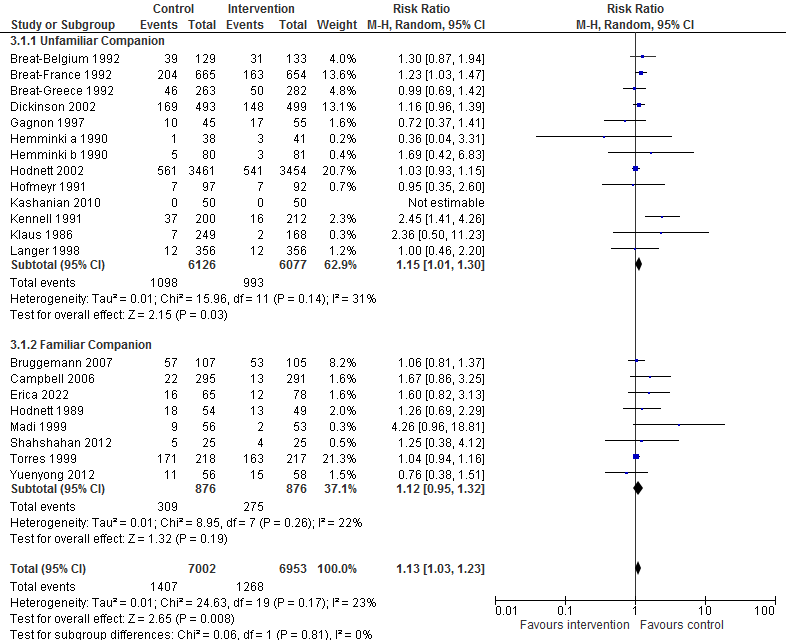
**

**Geographical region**


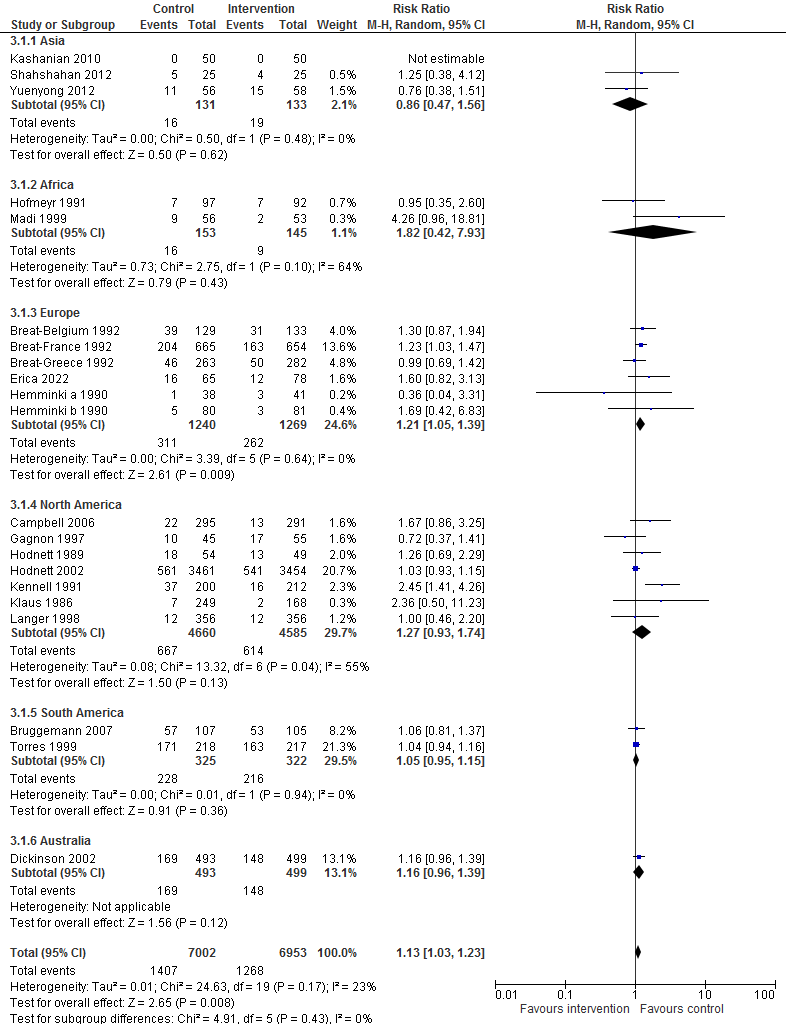

Supplement: S4 File — (DOCX) [file pone.0298852.s006.docx]
