# Supplementary material for: Impact of continuous labor companion- who is the best: A systematic review and meta-analysis of randomized controlled trials
Source: PLoS One. 2024 Jul 23;19(7):e0298852. doi: 10.1371/journal.pone.0298852 (PMC11265680; doi:10.1371/journal.pone.0298852)

**5 minute APGAR score < 7**


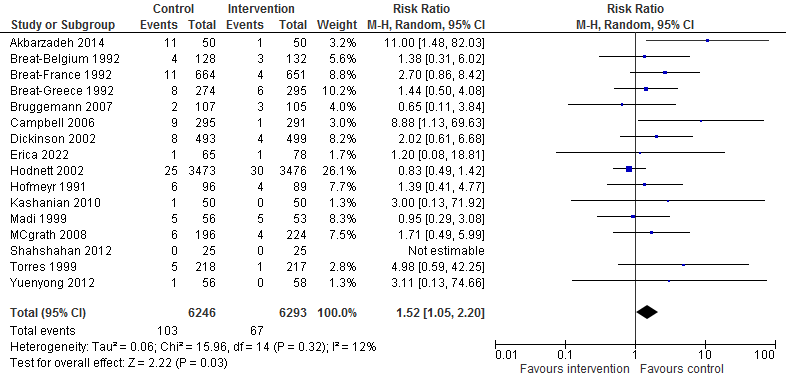


**Funnel Pot**


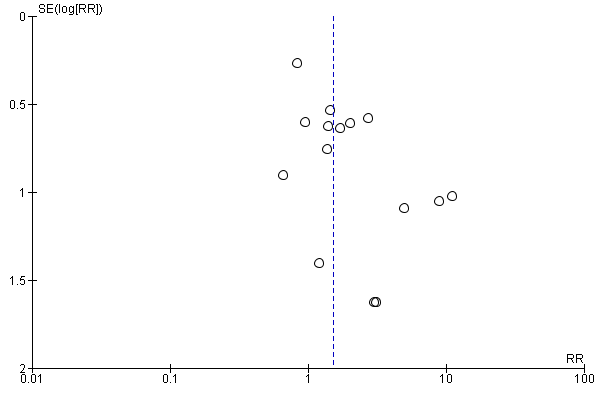


**Analysis of studies using a timeline as before and after 2000**


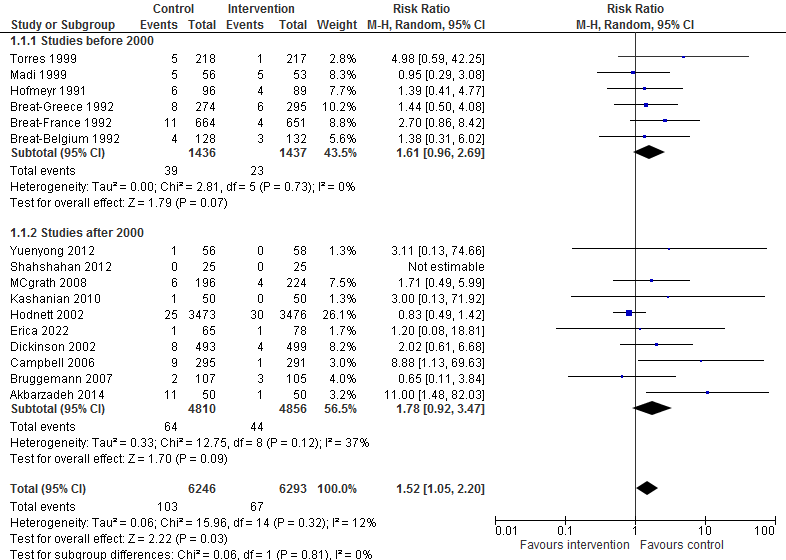


**Trained VS Untrained Labour Companion**


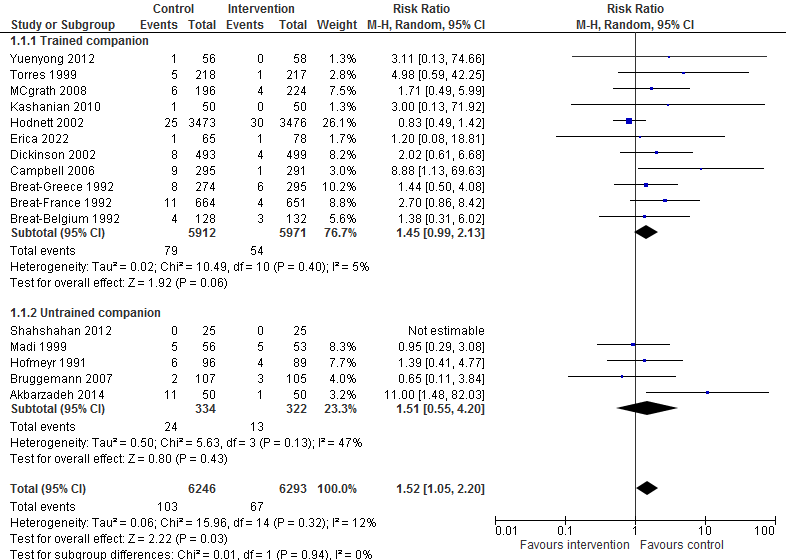


**Familiar vs Unfamiliar Labour Companion**


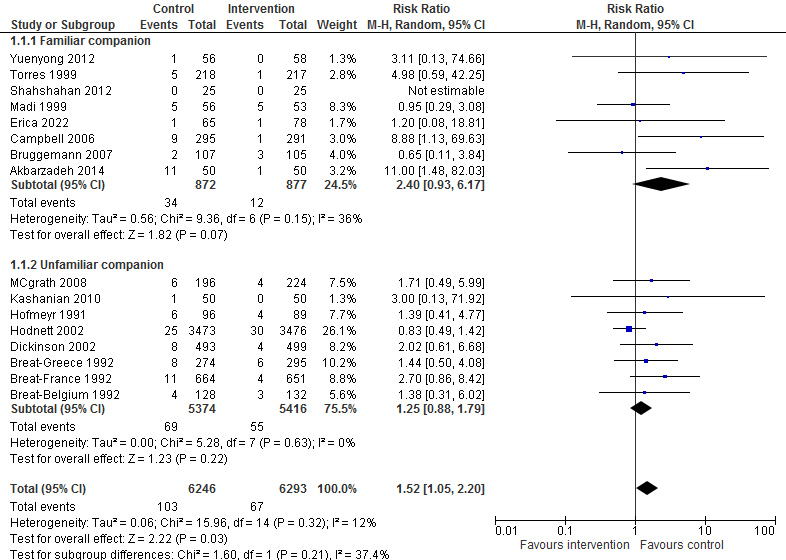


**Analysis by geographical region**


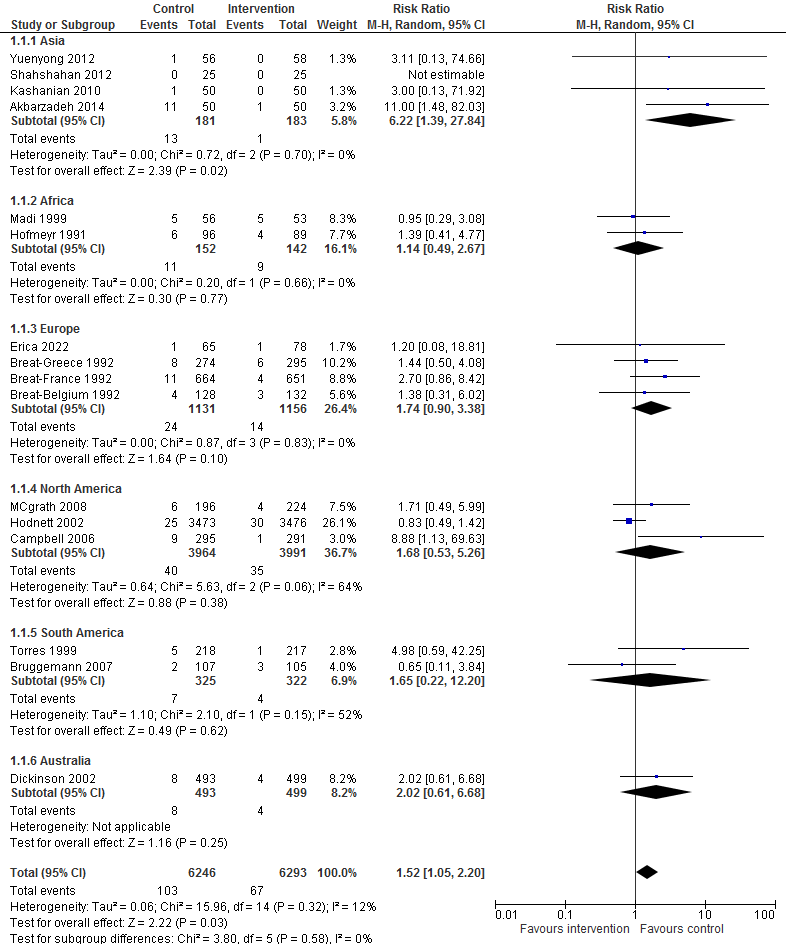

Supplement: S8 File — (DOCX) [file pone.0298852.s010.docx]
