# Supplementary material for: Impact of continuous labor companion- who is the best: A systematic review and meta-analysis of randomized controlled trials
Source: PLoS One. 2024 Jul 23;19(7):e0298852. doi: 10.1371/journal.pone.0298852 (PMC11265680; doi:10.1371/journal.pone.0298852)
Supplement: S10 File — (DOCX) [file pone.0298852.s012.docx]

**Search strategy**

We extracted Title-Abstract files in the formats of .csv, .ris, .bib, and .txt from the following databases to be exported and included in Rayyan.

1.PubMed

We conducted an advanced search on 04/07/2023. The results showed 158 articles.

((Labour companion [Title/Abstract]) OR (Birth partner [Title/Abstract]) OR (Doula [Title/Abstract]) OR (Labour support person [Title/Abstract]) OR (Childbirth coach [Title/Abstract]) OR (Labour assistant [Title/Abstract]) OR (Labour coach [Title/Abstract]) OR (Birth attendant [Title/Abstract]) OR (Labour caregiver [Title/Abstract]) OR (Maternity support person [Title/Abstract]) OR (Childbirth companion [Title/Abstract]) OR (Labour ally [Title/Abstract]) OR (Labour chaperon [Title/Abstract]))

AND

((Pregnancy outcome [Title/Abstract]) OR (Obstetric outcome [Title/Abstract]) OR (Delivery outcome [Title/Abstract]) OR (Birth outcome [Title/Abstract]) OR (Fetal outcome [Title/Abstract]) OR (Newborn outcome [Title/Abstract]) OR (Infant outcome [Title/Abstract]) OR (Neonatal outcome [Title/Abstract]) OR (Baby’s outcome [Title/Abstract]))

Result = 158 articles

2. Science Direct

We conducted an advanced search on 04/07/2023 using the following keywords combined with Boolean expressions. However, Science Direct allows a maximum of eight Boolean expressions only. Therefore, we modified the search terms and Boolean expressions used for other databases as follows. Under advanced search, we typed the following search terms combined with Boolean expressions in the section: ‘Title, abstract or author-specified keywords.’ The results showed 58 articles.

((Labour companion) OR (Doula) OR (Labour support person) OR (Childbirth companion) OR (Labour ally) OR (Labour chaperon))

AND

((Pregnancy outcome) OR (Birth outcome) OR (Newborn outcome))

Result = 58 articles

Then, we downloaded a hundred titles/abstracts at a time as RIS files and uploaded them into Rayyan.

3. Google Scholar

We conducted an advanced search on 04/07/2023 using the following keywords combined with Boolean expressions. We typed it in the section: ‘with all the words’ under advanced search. The results showed 466 articles.

((Labour companion) OR (Birth partner) OR (Doula) OR (Labour support person) OR (Childbirth coach) OR (Labour assistant) OR (Labour coach) OR (Birth attendant) OR (Labour caregiver) OR (Maternity support person) OR (Childbirth companion) OR (Labour ally) OR (Labour chaperon))

AND

((Pregnancy outcome) OR (Obstetric outcome) OR (Delivery outcome) OR (Birth outcome) OR (Fetal outcome) OR (Newborn outcome) OR (Infant outcome) OR (Neonatal outcome) OR (Baby’s outcome))

Result = 466 articles

Then we saved the articles to ‘My Library.’ After that, we used the ‘Export all’ option to get a CSV file to upload to Rayyan.

4. Research4life

We conducted an advanced search on 04/07/2023 using the following keywords combined with Boolean expressions. We typed it in the section: ‘What are you searching for?’ under advanced search. The initial results showed 2831 articles. Then we applied filters: Full Text Online, Scholarly and peer-reviewed, Journal Article, and English Language, resulting in 2766 articles.

((Labour companion) OR (Birth partner) OR (Doula) OR (Labour support person) OR (Childbirth coach) OR (Labour assistant) OR (Labour coach) OR (Birth attendant) OR (Labour caregiver) OR (Maternity support person) OR (Childbirth companion) OR (Labour ally) OR (Labour chaperon))

AND

((Pregnancy outcome) OR (Obstetric outcome) OR (Delivery outcome) OR (Birth outcome) OR (Fetal outcome) OR (Newborn outcome) OR (Infant outcome) OR (Neonatal outcome) OR (Baby’s outcome))

Result = 2766 articles

Then, we saved the articles to ‘Saved Items.’ After that, we used the ‘Export’ option to get a Bib Tex file to upload to Rayyan.

5. Cochrane Library

Date Run: 04/07/2023

We conducted an advanced search on 04/07/2023. We typed each search term under ‘search manager’ in the Cochrane database. We have reported results for each search term separately. Then, we combined the first thirteen search terms with the Boolean expression OR, resulting in 2303 articles. We combined search terms from fifteen to 23 using the same Boolean expression, resulting in 86100 articles. Finally, we combined results fourteen and twenty-four using the Boolean expression AND, resulting in 1765 articles.

ID Search Hits

#1 Birth Partner 1105

#2 Doula 113

#3 Labour companion 88

#4 Labour support person 543

#5 Childbirth coach 33

#6 Labour assistant 188

#7 Labour coach 46

#8 Birth attendant 282

#9 Labour caregiver 238

#10 Maternity support person 182

#11 Childbirth companion 64

#12 Labour ally 4

# 13 Labour chaperon 4

#14 #1 OR #2 OR #3 OR #4 OR #5 OR #6 OR #7 OR #8 OR #9 OR #10 OR #11 OR #12 OR #13 2303

#15 Pregnancy outcome 41713

#16 Obstetric outcome 6206

#17 Delivery outcome 31349

#18 Birth outcome 23646

#19 Fetal outcome 9580

#20 Newborn outcome 14860

#21 Infant outcome 24907

#22 Neonatal outcome 16647

#23 Baby’s outcome 4767

#24 #15 OR #16 OR #17 OR #18 OR #19 OR #20 OR #21 OR #22 OR #23 86100

#25 #14 AND #24 1765

6. Clinical Trials.gov

Date Run: 04/07/2023

((Labour companion) OR (Birth partner) OR (Doula) OR (Labour support person) OR (Childbirth coach) OR (Labour assistant) OR (Labour coach) OR (Birth attendant) OR (Labour caregiver) OR (Maternity support person) OR (Childbirth companion) OR (Labour ally) OR (Labour chaperon))

AND

((Pregnancy outcome) OR (Obstetric outcome) OR (Delivery outcome) OR (Birth outcome) OR (Fetal outcome) OR (Newborn outcome) OR (Infant outcome) OR (Neonatal outcome) OR (Baby’s outcome))

Result = 98 articles

7. ICTRP

Date Run: 04/07/2023

((Labour companion) OR (Birth partner) OR (Doula) OR (Labour support person) OR (Childbirth coach) OR (Labour assistant) OR (Labour coach) OR (Birth attendant) OR (Labour caregiver) OR (Maternity support person) OR (Childbirth companion) OR (Labour ally) OR (Labour chaperon))

AND

((Pregnancy outcome) OR (Obstetric outcome) OR (Delivery outcome) OR (Birth outcome) OR (Fetal outcome) OR (Newborn outcome) OR (Infant outcome) OR (Neonatal outcome) OR (Baby’s outcome))

Result = eight articles

8. Manual Search

We also conducted a manual search. Our selection criteria were the most cited, most recent ten meta-analyses in the same databases, searched using the same search strings. We searched 466 articles from Google Scholar, 58 articles from Science Direct, 1765 articles from the Cochrane database, 158 articles from PubMed, and 2766 articles from Research4life; the same articles were searched for RCTs. We did not search ICTRP or Clinical Trials.gov articles since those mainly contain individual trials but not meta-analyses. Altogether, we searched 5213 articles for meta-analyses.

After applying the predefined criteria, we selected one meta-analysis for manual search. It included 27 RCTs. Thus, we searched 27 articles using our manual search strategy.

One meta-analysis was selected.

(1) Ma B, Gj H, Sakala C, Rk F, Cuthbert A. Continuous support for women during childbirth ( Review ). 2017;(7).

Manual search = 27 studies
