# Supplementary material for: Impact of continuous labor companion- who is the best: A systematic review and meta-analysis of randomized controlled trials
Source: PLoS One. 2024 Jul 23;19(7):e0298852. doi: 10.1371/journal.pone.0298852 (PMC11265680; doi:10.1371/journal.pone.0298852)
Supplement: S11 File — (PDF) [file pone.0298852.s013.pdf]

INPLASY

# Impact of continuous labour companion- who is the best: A comprehensive meta-analysis on familiarity, training, temporal association, and geographical location

INPLASY202410003

doi: 10.37766/inplasy2024.1.0003

Received: 03 January 2024

Published: 03 January 2024

Jayasundara, DMCS[1]; Jayawardane, IA[2]; Weliange, SDS[3]; Jayasingha, TDKM[4]; Madugalle, TMSSB[5].

**Corresponding author:**
DMCS Jayasundara

chandana@obg.cmb.ac.lk

**Author Affiliation:**
Faculty of Medicine, University of Colombo, Sri Lanka.

**ADMINISTRATIVE INFORMATION**

**Support -** Self Funded.

**Review Stage at time of this submission -** Completed but not published.

**Conflicts of interest -** None declared.

**INPLASY registration number:** INPLASY202410003

**Amendments -** This protocol was registered with the International Platform of Registered Systematic Review and Meta-Analysis Protocols (INPLASY) on 03 January 2024 and was last updated on 03 January 2024.

## INTRODUCTION

**Review question / Objective** The present meta-analysis is aimed at describing the characteristics of the most effective labour companion, highlighting the differences in beneficial effects of having a labour companion among different geographical regions and timelines.

**Rationale** Having a companion for continuous labour support facilitates a smooth labour process, improving the maternal psychological status and fetal/neonatal well-being. Reported advantages include an increase in spontaneous vaginal births, reduced demand for analgesics, reduced need for oxytocin for labour augmentation, shorter duration of labour, decreased need for cesarean sections, minimal perineal trauma, and reduced requirement for instrumentation during labour, facilitating a smooth labour process (8–11). Maternal psychological well-being is improved by lowering tocophobia, postpartum depression, and anxiety and improving postpartum self-esteem and satisfaction (2,12,13). Fetal/neonatal well-being is enhanced by the early establishment of exclusive breastfeeding, early skin-to-skin contact, reduced neonatal hospital stay, and the need for neonatal resuscitation (14,15).

The quality of labour support and its beneficial outcomes depend on the type of companion used (16). The labour companion can be trained or untrained and familiar or unfamiliar to the parturient. The evidence regarding the type of best labour companion is controversial, and studies do not show a clear consensus. The rates of severe tocophobia, measured similarly, vary in different countries, and the reasons are unknown (17).

The prevalence of tokophobia was lower in the early years (1980s, 1990s) compared to more

Jayasundara et al. INPLASY protocol 202410003. doi:10.37766/inplasy2024.1.0003 Downloaded from https://inplasy.com/inplasy-2024-1-0003/

recent years (2000 onwards) (18). The beneficial effects of a labour companion can be more pronounced in some countries compared to others and in early years compared to more recent years.

**Condition being studied** The emotional process of labour and childbirth is often a fearful and stressful event for a pregnant mother (1). In most cultures, the tradition of supporting a woman in labour is a community event with multiple participants other than the designated healthcare provider. The fear and anxiety of childbirth are augmented by an unfamiliar hospital environment, medical jargon, procedures, interventions, and transient separation from the family during labour (2). The woman feels a sense of loss of control, isolation, and fear, peaking the level of anxiety (2). To cope with this tocophobia, pregnant women sometimes choose cesarean section over natural birth (3). Increased anxiety makes the woman more vulnerable to increased pain perception, prolonging the duration of labour and dystocia (4). The pain and anxiety during labour increase the endogenous catecholamine release, causing ineffective uterine contractions and decreased placental blood flow (5). An inefficient labour process may cause fetal and maternal complications, including the risk of fetal or neonatal hypoxia and death, infection, physical damage in the newborn, postpartum haemorrhage, maternal infection, and psychological distress due to anxiety, lack of sleep, and fatigue (4).
Different clinical settings have adopted strategies to alleviate tocophobia, facilitating a smooth labour process. These support methods include accompanying a companion for continuous labour support, induced sleep, hydrotherapy, and the Lamaze relaxation method (6). The labour companion can be a non-caregiving nurse, midwife, friend, relative, family member, husband, or a person trained in supporting labour (doula) (3). WHO defines Labour support as the supportive care provided to women during labour, including emotional support, physical comfort, advice, and information giving (5). WHO also recommends that a parturient should have a birth companion of her choice. However, it is not practised in many developing countries (7).

## METHODS

### Search strategy

We extracted Title-Abstract files in the formats of .csv, .ris, .bib, and .txt from the following databases to be exported and included in Rayyan.
1.PubMed
We conducted an advanced search on 04/07/2023. The results showed 158 articles.
((Labour companion [Title/Abstract]) OR (Birth partner [Title/Abstract]) OR (Doula [Title/Abstract]) OR (Labour support person [Title/Abstract]) OR (Childbirth coach [Title/Abstract]) OR (Labour assistant [Title/Abstract]) OR (Labour coach [Title/Abstract]) OR (Birth attendant [Title/Abstract]) OR (Labour caregiver [Title/Abstract]) OR (Maternity support person [Title/Abstract]) OR (Childbirth companion [Title/Abstract]) OR (Labour ally [Title/Abstract]) OR (Labour chaperon [Title/Abstract]))
AND
((Pregnancy outcome [Title/Abstract]) OR (Obstetric outcome [Title/Abstract]) OR (Delivery outcome [Title/Abstract]) OR (Birth outcome [Title/Abstract]) OR (Fetal outcome [Title/Abstract]) OR (Newborn outcome [Title/Abstract]) OR (Infant outcome [Title/Abstract]) OR (Neonatal outcome [Title/Abstract]) OR (Baby's outcome [Title/Abstract]))
Result = 158 articles
2. Science Direct
We conducted an advanced search on 04/07/2023 using the following keywords combined with Boolean expressions. However, Science Direct allows a maximum of eight Boolean expressions only. Therefore, we modified the search terms and Boolean expressions used for other databases as follows. Under advanced search, we typed the following search terms combined with Boolean expressions in the section: 'Title, abstract or author-specified keywords.' The results showed 58 articles.
((Labour companion) OR (Doula) OR (Labour support person) OR (Childbirth companion) OR (Labour ally) OR (Labour chaperon))
AND
((Pregnancy outcome) OR (Birth outcome) OR (Newborn outcome))
Result = 58 articles
Then, we downloaded a hundred titles/abstracts at a time as RIS files and uploaded them into Rayyan.
3. Google Scholar
We conducted an advanced search on 04/07/2023 using the following keywords combined with Boolean expressions. We typed it in the section: 'with all the words' under advanced search. The results showed 466 articles.
((Labour companion) OR (Birth partner) OR (Doula) OR (Labour support person) OR (Childbirth coach) OR (Labour assistant) OR (Labour coach) OR (Birth attendant) OR (Labour caregiver) OR (Maternity support person) OR (Childbirth companion) OR (Labour ally) OR (Labour chaperon))
AND
((Pregnancy outcome) OR (Obstetric outcome) OR (Delivery outcome) OR (Birth outcome) OR (Fetal outcome) OR (Newborn outcome) OR (Infant

Jayasundara et al. INPLASY protocol 202410003. doi:10.37766/inplasy2024.1.0003 Downloaded from https://inplasy.com/inplasy-2024-1-0003/

outcome) OR (Neonatal outcome) OR (Baby's outcome))
Result = 466 articles
Then we saved the articles to 'My Library.' After that, we used the 'Export all' option to get a CSV file to upload to Rayyan.
4. Research4life
We conducted an advanced search on 04/07/2023 using the following keywords combined with Boolean expressions. We typed it in the section: 'what are you searching for?' under advanced search. The initial results showed 2831 articles. Then we applied filters: Full Text Online, Scholarly & Peer-Reviewed, Journal Article and English Language resulting in 2766 articles.
((Labour companion) OR (Birth partner) OR (Doula) OR (Labour support person) OR (Childbirth coach) OR (Labour assistant) OR (Labour coach) OR (Birth attendant) OR (Labour caregiver) OR (Maternity support person) OR (Childbirth companion) OR (Labour ally) OR (Labour chaperon))
AND
((Pregnancy outcome) OR (Obstetric outcome) OR (Delivery outcome) OR (Birth outcome) OR (Fetal outcome) OR (Newborn outcome) OR (Infant outcome) OR (Neonatal outcome) OR (Baby's outcome))
Result = 2766 articles
Then we saved the articles to 'Saved Items.' After that, we used the 'Export' option to get a Bib Tex file to upload to Rayyan.
5. Cochrane Library
Date Run: 04/07/2023
ID Search Hits
#1 Birth Partner 1105
#2 Doula 113
#3 Labour companion 88
#4 Labour support person 543
#5 Childbirth coach 33
#6 Labour assistant 188
#7 Labour coach 46
#8 Birth attendant 282
#9 Labour caregiver 238
#10 Maternity support person 182
#11 Childbirth companion 64
#12 Labour ally 4
# 13 Labour chaperon 4
#14 #1 OR #2 OR #3 OR #4 OR #5 OR #6 OR #7 OR #8 OR #9 OR #10 OR #11 OR #12 OR #13 2303
#15 Pregnancy outcome 41713
#16 Obstetric outcome 6206
#17 Delivery outcome 31349
#18 Birth outcome 23646
#19 Fetal outcome 9580
#20 Newborn outcome 14860
#21 Infant outcome 24907
#22 Neonatal outcome 16647
#23 Baby's outcome 4767
#24 #15 OR #16 OR #17 OR #18 OR #19 OR #20 OR #21 OR #22 OR #23 86100
#25 #14 AND #24 1765
6. Clinical Trials.gov
Date Run: 04/07/2023
((Labour companion) OR (Birth partner) OR (Doula) OR (Labour support person) OR (Childbirth coach) OR (Labour assistant) OR (Labour coach) OR (Birth attendant) OR (Labour caregiver) OR (Maternity support person) OR (Childbirth companion) OR (Labour ally) OR (Labour chaperon))
AND
((Pregnancy outcome) OR (Obstetric outcome) OR (Delivery outcome) OR (Birth outcome) OR (Fetal outcome) OR (Newborn outcome) OR (Infant outcome) OR (Neonatal outcome) OR (Baby's outcome))
Result = 98 articles
7. ICTRP
Date Run: 04/07/2023
((Labour companion) OR (Birth partner) OR (Doula) OR (Labour support person) OR (Childbirth coach) OR (Labour assistant) OR (Labour coach) OR (Birth attendant) OR (Labour caregiver) OR (Maternity support person) OR (Childbirth companion) OR (Labour ally) OR (Labour chaperon))
AND
((Pregnancy outcome) OR (Obstetric outcome) OR (Delivery outcome) OR (Birth outcome) OR (Fetal outcome) OR (Newborn outcome) OR (Infant outcome) OR (Neonatal outcome) OR (Baby's outcome))
Result = 8 articles.
8. Manual Search
We also conducted a manual search. Our selection criteria were the most cited, most recent ten meta-analyses in the same databases, searched using the same search strings. We searched 466 articles from Google Scholar, 58 articles from Science Direct, 1765 articles from the Cochrane database, 158 articles from PubMed, and 2766 articles from Research4life which were the same articles searched for RCTs. We didn't search ICTRP or Clinical Trials.gov articles since those mainly contain individual trials but not meta-analyses. Altogether, we searched 5213 articles for meta-analyses.
After applying the predefined criteria, we selected one meta-analysis for manual search. It included 27 RCTs. Thus, we searched 27 articles using our manual search strategy.
One meta-analysis was selected.
(1) Ma B, Gj H, Sakala C, Rk F, Cuthbert A. Continuous support for women during childbirth ( Review ). 2017;(7).
Manual search = 27 studies

Jayasundara et al. INPLASY protocol 202410003. doi:10.37766/inplasy2024.1.0003 Downloaded from https://inplasy.com/inplasy-2024-1-0003/

**Participant or population** Low-risk women with a single fetus in cephalic presentation, admitted during early labour (cervical dilation 3-4 cm) with no contraindications for vaginal delivery.

**Intervention** A companion for continuous labour support.

**Comparator** Routine labour care without a companion for continuous labour support.

**Study designs to be included** Randomised Controlled Trials.

**Eligibility criteria** Randomised controlled trials (RCTs) with full-text articles reporting results related to low-risk women with a single fetus in cephalic presentation, admitted during early labour (cervical dilation 3-4 cm) with no contraindications for vaginal delivery were included in the study. Studies reporting women with medical or psychiatric diseases, previous cesarean section, genital abnormalities, fetal distress, and any fetal anomaly and review articles, case reports, documents, or observational studies were excluded.

**Information sources** PubMed, Science Direct, Cochrane Library, Google Scholar, ClinicalTrials.gov, and International Clinical Trials Registry Platform (ICTRP) were searched. A manual search strategy was also applied to ensure inclusivity, focusing on identifying any missing studies by reviewing the most cited ten meta-analyses within the same databases.

**Main outcome(s)** Reporting meta-analyses of 8 outcomes:
1. Spontaneous vaginal delivery
2. Duration of labour hr (Standard mean difference)
3. Cesarean section
4. Instrumental delivery
5. Oxytocin for labour induction
6. Analgesic usage
7. Tocophobia
8. 5 min APGAR < 7

, as risk ratios and standard mean differences with 95% confidence intervals. Here overall effects were reported without subgroup analyses.

**Additional outcome(s)** Subgroup analyses were conducted related to main outcomes to compare the effects of trained vs. untrained labour companions, familiar vs. unfamiliar labour companions, studies before vs. after 2000, and studies in different geographical locations.

**Data management** We extracted Title-Abstract files in the formats of .csv, .ris, .bib, and .txt from the electronic databases to be exported and included in Rayyan. The study selection process was carried out meticulously in two rounds using a semi-automated tool called Rayyan (19), with one author as the reviewer and another as a collaborator, employing a blind approach. In the first round, titles and abstracts were screened, eliminating duplicates and ineligible entries, with conflicts resolved by the reviewer. The second round involved a similar blind approach for full-text screening, again with conflicts resolved by the reviewer. The authors were contacted in cases requiring additional information, and the study selection process was transparently reported using the PRISMA 2020 flow diagram for updated systematic reviews (20).
Key study characteristics were extracted and organised into predefined tables for outcome measures concerning the facilitation of the labour process, maternal psychological well-being, and fetal well-being. Concerned outcome measures were Spontaneous vaginal birth, Tocophobia, Postpartum depression, Admission to a special care nursery, Exclusive breastfeeding, Analgesic usage, Synthetic oxytocin usage, duration of labour, LSCS rate, Labour pain, Instrumental vaginal delivery, Perineal trauma, 5-min APGAR score, Neonatal hospital stay, Maternal anxiety, Maternal self-esteem, and maternal satisfaction. To ensure the integrity of the research, a second author independently reviewed the entire process, minimising the potential for bias.

**Quality assessment / Risk of bias analysis** The quality of each RCT was assessed using the Cochrane risk-of-bias (RoB2) tool (21). Random sequence generation, allocation concealment, performance bias, detection bias, attrition bias, reporting bias, and other biases are used as criteria in RoB2. Funnel plots were employed to gauge publication bias, with any deviation from the expected funnel-shaped distribution as an indicator of potential publication bias.

**Strategy of data synthesis** We used RevMan version 5.4 to analyse the following outcome measures reported by more than ten RCTs - Spontaneous vaginal birth, Tocophobia, Use of analgesics, Need for synthetic oxytocin, duration of labour, LSCS rate, Instrumental vaginal delivery, and 5-min APGAR score. The Mantel-Haenszel statistical method, random effects analysis model, and risk ratio with 95% confidence interval (CI) as effect measure were used for dichotomous data. For the continuous data inverse variance statistical method, the random effects analysis model and

Jayasundara et al. INPLASY protocol 202410003. doi:10.37766/inplasy2024.1.0003 Downloaded from https://inplasy.com/inplasy-2024-1-0003/

standard mean difference as effect measures were used. We assessed heterogeneity with the I2 statistic, considering p value 50% indicators of significant heterogeneity.

**Subgroup analysis** Subgroup analyses were conducted using RevMan 5.4 to compare the effects of trained vs. untrained labour companions, familiar vs. unfamiliar labour companions, studies before vs. after 2000, and studies in different geographical locations.

**Sensitivity analysis** It is not possible to conduct sensitivity analyses in RevMan by omitting individual diagnostic studies.

**Language restriction** RCTs published in English language.

**Country(ies) involved** Sri Lanka.

**Keywords** Continuous labour companion, Meta-analysis, Trained companion, Untrained companion, Familiar companion, Unfamiliar companion, Temporal association, Asia, Africa, Europe.

**Dissemination plans** The study will be published in a peer reviewed, indexed journal.

**Contributions of each author**

Author 1 - DMCS Jayasundara - • The author contributed in; • Design: Directly involved in design of the study • Planning: Directly involved in planning of the study • Conduct: Title: abstract screening using Rayyan. • Data analysis: Interpretation of analyzed data in practical obstetric point of view. • Manuscript writing.
Email: chandana@obg.cmb.ac.lk

Author 2 - IA Jayawardane - • The author contributed in; • Design: Directly involved in design of the study • Planning: Directly involved in planning of the study • Conduct: Extraction of individual study data into predefined tables. • Data analysis: Interpretation of analyzed data in practical obstetric point of view. • Manuscript writing.
Email: asanka@obg.cmb.ac.lk

Author 3 - SDS Weliange - • The author contributed in; • Design: Directly involved in design of the study • Planning: Directly involved in planning of the study • Conduct: Downloaded full text articles and uploaded to Rayyan. • Data analysis: Interpretation of analyzed data in statistical point of view. • Manuscript writing.
Email: shreenika@commed.cmb.ac.lk

Author 4 - TDKM Jayasingha - • The author contributed in; • Design: Directly involved in design of the study • Planning: Directly involved in planning of the study • Conduct: Abstract screening using Rayyan. • Data analysis: Analyzed data using RevMan. • Manuscript writing.
Email: kavindamadushan1134@gmail.com

Author 5 - TMSSB Madugalle - • The author contributed in; • Design: Directly involved in design of the study • Planning: Directly involved in planning of the study • Conduct: Extraction of individual study data into predefined tables. • Data analysis: Interpretation of analyzed data in practical obstetric point of view. • Manuscript writing.
Email: madugallesameera@gmail.com

Jayasundara et al. INPLASY protocol 202410003. doi:10.37766/inplasy2024.1.0003 Downloaded from https://inplasy.com/inplasy-2024-1-0003/
