# Supplementary material for: Impact of continuous labor companion- who is the best: A systematic review and meta-analysis of randomized controlled trials
Source: PLoS One. 2024 Jul 23;19(7):e0298852. doi: 10.1371/journal.pone.0298852 (PMC11265680; doi:10.1371/journal.pone.0298852)
Supplement: S12 File — (DOCX) [file pone.0298852.s014.docx]

**Outcome 1: Spontaneous vaginal birth**

| Study | Intervention | Control |
| --- | --- | --- |
| Ana Langer-Mexico(1998) | 260/357 | 247/357 |
| Anita J Gagnon-Canada(1997) |  |  |
| Banyana Cecilia Madi-Botswana(1999) | 48/53 | 40/56 |
| E Hemminki-Finland(1990) | 1. 38/41, b- 76/81 | a-34/38, b-72/80 |
| Ellen D Hodnett-Canada(1989) | 2481/3454 | 2463/3461 |
| G Justus Hofmeyr-South Africa(1991) | 74/92 | 76/97 |
| John Kennel-US(1991) | 179/212 | 137/200 |
| Marshal H Klaus-Gautemala(1986) | 154/168 | 196/249 |
| Rosemary Cogan-US(1988) |  |  |
| Coralie Trotter-South Africa(1992) |  |  |
| V Cheryl Nikodem-South Africa(1998) |  |  |
| Wendy Lynne Wolman-South Africa(1993) |  |  |
|  |  |  |
| Bruggemann-Brazil(2007) | 41/105 | 38/107 |
| Campbell-US(2006) | 223/291 | 220/295 |
| Hodnett-US&Canada(2002) |  |  |
| MCgrath-US(2008) |  |  |
| Morhason Bello-Nigeria(2009) |  |  |
| Campbell-US(2007) |  |  |
| Guadalupe Trueba-Mexico(2000) |  |  |
|  |  |  |
| Isbir-Turkey(2015) |  |  |
| Kashanian-Iran(2010) | 46/50 | 38/50 |
| Safarzadeh-Iran(2012) | 73/75 | 74/75 |
| Yuenyong-Thailand(2012) | 33/58 | 31/58 |
| Aida Aalrazek-Jordan(2012) |  |  |
| Zahra Shahshahan-Iran(2012) |  |  |
|  |  |  |
| Akira Shibanuma-Ghana(2021) |  |  |
| Atefeh Kazemi Robati-Iran(2020) |  |  |
| Atefeh Salehi-Iran(2016) |  |  |
| Erica Schytt-Sweden(2022) |  |  |
| Nahid Bolbol-Iran(2016) |  |  |
| Breat-Belgium (1992) | 97/133 | 85/129 |
| Breat-France (1992) | 451/654 | 425/665 |
| Breat-Greece (1992) | 202/282 | 183/263 |
| Torres – Chile (1999) | 110/217 | 101/218 |
| Dickinson-Australia (2002) | 280/499 | 239/493 |
| Akbarzadeh-Iran (2014) | 47/50 | 30/50 |

**Outcome 2: Analgesic usage**

| Study | Intervention | Control |
| --- | --- | --- |
| Ana Langer-Mexico(1998) | 302/346 | 295/335 |
| Anita J Gagnon-Canada(1997) | 33/45 | 37/55 |
| Banyana Cecilia Madi-Botswana(1999) | 41/56 | 28/53 |
| E Hemminki-Finland(1990) | a-23/38  b-52/80 | a-25/41  b-45/81 |
| Ellen D Hodnett-Canada(1989) | 44/54 | 30/49 |
| G Justus Hofmeyr-South Africa(1991) | 56/97 | 52/92 |
| John Kennel-US(1991) |  |  |
| Marshal H Klaus-Gautemala(1986) | 10/249 | 2/168 |
| Rosemary Cogan-US(1988) |  |  |
| Coralie Trotter-South Africa(1992) |  |  |
| V Cheryl Nikodem-South Africa(1998) |  |  |
| Wendy Lynne Wolman-South Africa(1993) |  |  |
|  |  |  |
| Bruggemann-Brazil(2007) |  |  |
| Campbell-US(2006) | 260/295 | 247/291 |
| Hodnett-US&Canada(2002) | 2436/3461 | 2349/3454 |
| MCgrath-US(2008) | 149/196 | 145/224 |
| Morhason Bello-Nigeria(2009) | 89/292 | 84/293 |
| Campbell-US(2007) |  |  |
| Guadalupe Trueba-Mexico(2000) | 16/50 | 4/50 |
|  |  |  |
| Isbir-Turkey(2015) |  |  |
| Kashanian-Iran(2010) |  |  |
| Safarzadeh-Iran(2012) |  |  |
| Yuenyong-Thailand(2012) | 15/56 | 13/58 |
| Aida Aalrazek-Jordan(2012) |  |  |
| Zahra Shahshahan-Iran(2012) |  |  |
|  |  |  |
| Akira Shibanuma-Ghana(2021) |  |  |
| Atefeh Kazemi Robati-Iran(2020) |  |  |
| Atefeh Salehi-Iran(2016) |  |  |
| Erica Schytt-Sweden(2022) | 24/65 | 31/78 |
| Nahid Bolbol-Iran(2016) |  |  |
| Breat-Belgium (1992) | 62/131 | 55/133 |
| Breat-France (1992) | 319/666 | 281/652 |
| Breat-Greece (1992) |  |  |
| Torres – Chile (1999) | 195/218 | 202/217 |
| Dickinson-Australia (2002) |  |  |
| Akbarzadeh-Iran (2014) |  |  |

**Outcome 3: Synthetic oxytocin during labour**

| Study | Intervention | Control |
| --- | --- | --- |
| Ana Langer-Mexico(1998) |  |  |
| Anita J Gagnon-Canada(1997) |  |  |
| Banyana Cecilia Madi-Botswana(1999) | 7/53 | 17/56 |
| E Hemminki-Finland(1990) | a-3/41  b-8/81 | a-3/38  b-16/80 |
| Ellen D Hodnett-Canada(1989) | 5/49 | 16/49 |
| G Justus Hofmeyr-South Africa(1991) | 16/92 | 17/97 |
| John Kennel-US(1991) | 36/212 | 46/200 |
| Marshal H Klaus-Gautemala(1986) | 4/168 | 33/249 |
| Rosemary Cogan-US(1988) |  |  |
| Coralie Trotter-South Africa(1992) |  |  |
| V Cheryl Nikodem-South Africa(1998) |  |  |
| Wendy Lynne Wolman-South Africa(1993) |  |  |
|  |  |  |
| Bruggemann-Brazil(2007) | 104/105 | 107/107 |
| Campbell-US(2006) | 133/291 | 144/295 |
| Hodnett-US&Canada(2002) | 1040/3454 | 942/3461 |
| MCgrath-US(2008) |  |  |
| Morhason Bello-Nigeria(2009) | 51/293 | 56/292 |
| Campbell-US(2007) |  |  |
| Guadalupe Trueba-Mexico(2000) |  |  |
|  |  |  |
| Isbir-Turkey(2015) | 23/33 | 25/30 |
| Kashanian-Iran(2010) | 11/50 | 19/50 |
| Safarzadeh-Iran(2012) | 18/75 | 19/75 |
| Yuenyong-Thailand(2012) | 21/58 | 18/56 |
| Aida Aalrazek-Jordan(2012) |  |  |
| Zahra Shahshahan-Iran(2012) |  |  |
|  |  |  |
| Akira Shibanuma-Ghana(2021) |  |  |
| Atefeh Kazemi Robati-Iran(2020) |  |  |
| Atefeh Salehi-Iran(2016) |  |  |
| Erica Schytt-Sweden(2022) |  |  |
| Nahid Bolbol-Iran(2016) | 7/50 | 8/50 |
| Breat-Belgium (1992) | 55/132 | 64/129 |
| Breat-France (1992) | 383/654 | 371/666 |
| Breat-Greece (1992) | 224/287 | 193/265 |
| Torres – Chile (1999) | 21/50 | 48/50 |
| Dickinson-Australia (2002) |  |  |
| Akbarzadeh-Iran (2014) |  |  |

**Outcome 4: Labour length (Hours)**

| Study | Intervention | Control |
| --- | --- | --- |
| Ana Langer-Mexico(1998) | 4.56+/-3.47(n=361) | 5.58+/-3.47(n=363) |
| Anita J Gagnon-Canada(1997) | 8.9+/-3.7(n=55) | 9.6+/-4.9(n=45) |
| Banyana Cecilia Madi-Botswana(1999) |  |  |
| E Hemminki-Finland(1990) | a-8.3+/-6.2(n=41), b-7.6+/-4.9(n=81) | a-10+/-6.8(n=38)  b-9.2+/-6.1(n=80) |
| Ellen D Hodnett-Canada(1989) |  |  |
| G Justus Hofmeyr-South Africa(1991) | 9.6+/-3.92(n=92) | 10.2+/-4.96(n=97) |
| John Kennel-US(1991) | 7.4+/-3.8(n=212) | 8.4+/-4.2(n=200) |
| Marshal H Klaus-Gautemala(1986) |  |  |
| Rosemary Cogan-US(1988) | 10.7+/-5.66(n=14) | 20.9+/-18.87(n=11) |
| Coralie Trotter-South Africa(1992) |  |  |
| V Cheryl Nikodem-South Africa(1998) |  |  |
| Wendy Lynne Wolman-South Africa(1993) |  |  |
|  |  |  |
| Bruggemann-Brazil(2007) |  |  |
| Campbell-US(2006) | 10.4+/-4.3(n=291) | 11.7+/-4.8(n=295) |
| Hodnett-US&Canada(2002) |  |  |
| MCgrath-US(2008) |  |  |
| Morhason Bello-Nigeria(2009) | 4.7+/-1.7(n=293) | 5.3+/-1.7(n=292) |
| Campbell-US(2007) |  |  |
| Guadalupe Trueba-Mexico(2000) | 14.5+/-5.36(n=50) | 19.38+/-7.3(n=50) |
|  |  |  |
| Isbir-Turkey(2015) | 8+/-3.1(n=33) | 12.7+/-5(n=30) |
| Kashanian-Iran(2010) |  |  |
| Safarzadeh-Iran(2012) |  |  |
| Yuenyong-Thailand(2012) | 11.82+/-5.6(n=58) | 12.48+/-7.85(n=56) |
| Aida Aalrazek-Jordan(2012) |  |  |
| Zahra Shahshahan-Iran(2012) | 5.28+/-0.83(n=25) | 5.78+/-1.12(n=25) |
|  |  |  |
| Akira Shibanuma-Ghana(2021) |  |  |
| Atefeh Kazemi Robati-Iran(2020) |  |  |
| Atefeh Salehi-Iran(2016) |  |  |
| Erica Schytt-Sweden(2022) |  |  |
| Nahid Bolbol-Iran(2016) | 8.77+/-3.58(n=50) | 12.53+/-3.76(n=50) |
| Breat-Belgium (1992) | 6.27+/-5.37(n=133) | 6.8+/-4.07(n=129) |
| Breat-France (1992) | 6.77+/-2.57(n=654) | 7.07+/-2.68(n=666) |
| Breat-Greece (1992) | 6.67+/-2.57(n=654) | 6.33+/-3.92(n=265) |
| Torres – Chile (1999) |  |  |
| Dickinson-Australia (2002) |  |  |
| Akbarzadeh-Iran (2014) |  |  |

**Outcome 5: Cesarean birth**

| Study | Intervention | Control |
| --- | --- | --- |
| Ana Langer-Mexico(1998) | 85/357 | 97/356 |
| Anita J Gagnon-Canada(1997) | 7/55 | 13/45 |
| Banyana Cecilia Madi-Botswana(1999) | 3/53 | 7/56 |
| E Hemminki-Finland(1990) | a-0/41  b-2/81 | a-3/38  b-3/80 |
| Ellen D Hodnett-Canada(1989) |  |  |
| G Justus Hofmeyr-South Africa(1991) | 11/92 | 14/97 |
| John Kennel-US(1991) | 17/212 | 26/200 |
| Marshal H Klaus-Gautemala(1986) | 11/168 | 43/249 |
| Rosemary Cogan-US(1988) |  |  |
| Coralie Trotter-South Africa(1992) |  |  |
| V Cheryl Nikodem-South Africa(1998) |  |  |
| Wendy Lynne Wolman-South Africa(1993) |  |  |
|  |  |  |
| Bruggemann-Brazil(2007) | 11/105 | 12/107 |
| Campbell-US(2006) | 55/291 | 53/295 |
| Hodnett-US&Canada(2002) | 432/3454 | 437/3461 |
| MCgrath-US(2008) | 30/224 | 49/196 |
| Morhason Bello-Nigeria(2009) | 24/293 | 65/292 |
| Campbell-US(2007) |  |  |
| Guadalupe Trueba-Mexico(2000) | 1/50 | 12/50 |
|  |  |  |
| Isbir-Turkey(2015) | 3/36 | 6/36 |
| Kashanian-Iran(2010) | 4/50 | 12/50 |
| Safarzadeh-Iran(2012) |  |  |
| Yuenyong-Thailand(2012) | 10/58 | 14/56 |
| Aida Aalrazek-Jordan(2012) |  |  |
| Zahra Shahshahan-Iran(2012) |  |  |
|  |  |  |
| Akira Shibanuma-Ghana(2021) |  |  |
| Atefeh Kazemi Robati-Iran(2020) |  |  |
| Atefeh Salehi-Iran(2016) |  |  |
| Erica Schytt-Sweden(2022) | 9/78 | 12/65 |
| Nahid Bolbol-Iran(2016) |  |  |
| Breat-Belgium (1992) | 5/133 | 5/129 |
| Breat-France (1992) | 40/654 | 36/665 |
| Breat-Greece (1992) | 30/282 | 34/263 |
| Torres – Chile (1999) | 54/217 | 46/218 |
| Dickinson-Australia (2002) | 71/499 | 85/493 |
| Akbarzadeh-Iran (2014) | 3/50 | 20/50 |

**Outcome 6: Instrumental vaginal birth**

| Study | Intervention | Control |
| --- | --- | --- |
| Ana Langer-Mexico(1998) | 12/356 | 12/356 |
| Anita J Gagnon-Canada(1997) | 17/55 | 10/45 |
| Banyana Cecilia Madi-Botswana(1999) | 2/53 | 9/56 |
| E Hemminki-Finland(1990) | a-3/41  b-3-81 | a-1/38  b-5/80 |
| Ellen D Hodnett-Canada(1989) | 13/49 | 18/54 |
| G Justus Hofmeyr-South Africa(1991) | 7/92 | 7/97 |
| John Kennel-US(1991) | 16/212 | 37/200 |
| Marshal H Klaus-Gautemala(1986) | 2/168 | 7/249 |
| Rosemary Cogan-US(1988) |  |  |
| Coralie Trotter-South Africa(1992) |  |  |
| V Cheryl Nikodem-South Africa(1998) |  |  |
| Wendy Lynne Wolman-South Africa(1993) |  |  |
|  |  |  |
| Bruggemann-Brazil(2007) | 53/105 | 57/107 |
| Campbell-US(2006) | 13/291 | 22/295 |
| Hodnett-US&Canada(2002) | 541/3454 | 561/3461 |
| MCgrath-US(2008) |  |  |
| Morhason Bello-Nigeria(2009) |  |  |
| Campbell-US(2007) |  |  |
| Guadalupe Trueba-Mexico(2000) |  |  |
|  |  |  |
| Isbir-Turkey(2015) |  |  |
| Kashanian-Iran(2010) | 0/50 | 0/50 |
| Safarzadeh-Iran(2012) |  |  |
| Yuenyong-Thailand(2012) | 15/58 | 11/56 |
| Aida Aalrazek-Jordan(2012) |  |  |
| Zahra Shahshahan-Iran(2012) | 4/25 | 5/25 |
|  |  |  |
| Akira Shibanuma-Ghana(2021) |  |  |
| Atefeh Kazemi Robati-Iran(2020) |  |  |
| Atefeh Salehi-Iran(2016) |  |  |
| Erica Schytt-Sweden(2022) | 12/78 | 16/65 |
| Nahid Bolbol-Iran(2016) |  |  |
| Breat-Belgium (1992) | 31/133 | 39/129 |
| Breat-France (1992) | 163/654 | 204/665 |
| Breat-Greece (1992) | 50/282 | 46/263 |
| Torres – Chile (1999) | 163/217 | 171/218 |
| Dickinson-Australia (2002) | 148/499 | 169/493 |
| Akbarzadeh-Iran (2014) |  |  |

**Outcome 7: Five min Apgar score < 7**

| Study | Intervention | Control |
| --- | --- | --- |
| Ana Langer-Mexico(1998) |  |  |
| Anita J Gagnon-Canada(1997) |  |  |
| Banyana Cecilia Madi-Botswana(1999) | 5/53 | 5/56 |
| E Hemminki-Finland(1990) |  |  |
| Ellen D Hodnett-Canada(1989) |  |  |
| G Justus Hofmeyr-South Africa(1991) | 4/89 | 6/96 |
| John Kennel-US(1991) |  |  |
| Marshal H Klaus-Gautemala(1986) |  |  |
| Rosemary Cogan-US(1988) |  |  |
| Coralie Trotter-South Africa(1992) |  |  |
| V Cheryl Nikodem-South Africa(1998) |  |  |
| Wendy Lynne Wolman-South Africa(1993) |  |  |
|  |  |  |
| Bruggemann-Brazil(2007) | 3/105 | 2/107 |
| Campbell-US(2006) | 1/291 | 9/295 |
| Hodnett-US&Canada(2002) | 30/3476 | 25/3473 |
| MCgrath-US(2008) | 4/224 | 6/196 |
| Morhason Bello-Nigeria(2009) |  |  |
| Campbell-US(2007) |  |  |
| Guadalupe Trueba-Mexico(2000) |  |  |
|  |  |  |
| Isbir-Turkey(2015) |  |  |
| Kashanian-Iran(2010) | 0/50 | 1/50 |
| Safarzadeh-Iran(2012) |  |  |
| Yuenyong-Thailand(2012) | 0/58 | 1/56 |
| Aida Aalrazek-Jordan(2012) |  |  |
| Zahra Shahshahan-Iran(2012) | 0/25 | 0/25 |
|  |  |  |
| Akira Shibanuma-Ghana(2021) |  |  |
| Atefeh Kazemi Robati-Iran(2020) |  |  |
| Atefeh Salehi-Iran(2016) |  |  |
| Erica Schytt-Sweden(2022) | 1/78 | 1/65 |
| Nahid Bolbol-Iran(2016) |  |  |
| Breat-Belgium (1992) | 3/132 | 4/128 |
| Breat-France (1992) | 4/651 | 11/664 |
| Breat-Greece (1992) | 6/295 | 8/274 |
| Torres – Chile (1999) | 1/217 | 5/218 |
| Dickinson-Australia (2002) | 4/499 | 8/493 |
| Akbarzadeh-Iran (2014) | 1/50 | 11/50 |

**Outcome 8: Tocophobia**

| Study | Intervention | Control |
| --- | --- | --- |
| Ana Langer-Mexico(1998) | 98/357 | 129/353 |
| Anita J Gagnon-Canada(1997) |  |  |
| Banyana Cecilia Madi-Botswana(1999) |  |  |
| E Hemminki-Finland(1990) |  |  |
| Ellen D Hodnett-Canada(1989) |  |  |
| G Justus Hofmeyr-South Africa(1991) | 38/92 | 73/96 |
| John Kennel-US(1991) | 47/209 | 71/197 |
| Marshal H Klaus-Gautemala(1986) |  |  |
| Rosemary Cogan-US(1988) |  |  |
| Coralie Trotter-South Africa(1992) |  |  |
| V Cheryl Nikodem-South Africa(1998) |  |  |
| Wendy Lynne Wolman-South Africa(1993) |  |  |
|  |  |  |
| Bruggemann-Brazil(2007) | 7/105 | 17/107 |
| Campbell-US(2006) | 95/229 | 197/265 |
| Hodnett-US&Canada(2002) | 96/2818 | 117/2751 |
| MCgrath-US(2008) |  |  |
| Morhason Bello-Nigeria(2009) | 108/293 | 196/292 |
| Campbell-US(2007) |  |  |
| Guadalupe Trueba-Mexico(2000) |  |  |
|  |  |  |
| Isbir-Turkey(2015) |  |  |
| Kashanian-Iran(2010) |  |  |
| Safarzadeh-Iran(2012) |  |  |
| Yuenyong-Thailand(2012) |  |  |
| Aida Aalrazek-Jordan(2012) |  |  |
| Zahra Shahshahan-Iran(2012) |  |  |
|  |  |  |
| Akira Shibanuma-Ghana(2021) |  |  |
| Atefeh Kazemi Robati-Iran(2020) |  |  |
| Atefeh Salehi-Iran(2016) |  |  |
| Erica Schytt-Sweden(2022) |  |  |
| Nahid Bolbol-Iran(2016) |  |  |
| Breat-Belgium (1992) | 24/119 | 30/121 |
| Breat-France (1992) | 30/656 | 35/664 |
| Breat-Greece (1992) |  |  |
| Torres – Chile (1999) | 35/206 | 43/211 |
| Dickinson-Australia (2002) | 75/499 | 74/493 |
| Akbarzadeh-Iran (2014) |  |  |

**Type of companion**

| Study | Intervention |
| --- | --- |
| Ana Langer-Mexico(1998) | Female companion |
| Anita J Gagnon-Canada(1997) | Nurses |
| Banyana Cecilia Madi-Botswana(1999) | Female relative |
| E Hemminki-Finland(1990) | Midwifery students |
| Ellen D Hodnett-Canada(1989) | Self-employed birth attendants- familiar with mother |
| G Justus Hofmeyr-South Africa(1991) | Female volunteers from community- no experience |
| John Kennel-US(1991) | Trained unfamiliar females |
| Marshal H Klaus-Gautemala(1986) | Lay Guatemala women |
| Rosemary Cogan-US(1988) | Unrelated female trained supporter |
| Coralie Trotter-South Africa(1992) | Untrained community doula(female) |
| V Cheryl Nikodem-South Africa(1998) | Untrained community doula(female) |
| Wendy Lynne Wolman-South Africa(1993) |  |
|  |  |
| Bruggemann-Brazil(2007) | Companion of choice |
| Campbell-US(2006) | Trained lay doula |
| Hodnett-US&Canada(2002) | Nurses |
| MCgrath-US(2008) | Trained female doula |
| Morhason Bello-Nigeria(2009) | Companion of choice |
| Campbell-US(2007) | Trained lay doula |
| Guadalupe Trueba-Mexico(2000) | Trained doula |
|  |  |
| Isbir-Turkey(2015) | Midwifery students |
| Kashanian-Iran(2010) | Experienced midwife |
| Safarzadeh-Iran(2012) | Female friend/relative |
| Yuenyong-Thailand(2012) | Trained close female relative |
| Aida Aalrazek-Jordan(2012) | Trained significance others choice(educated female) |
| Zahra Shahshahan-Iran(2012) | Support person |
|  |  |
| Akira Shibanuma-Ghana(2021) |  |
| Atefeh Kazemi Robati-Iran(2020) | Midwife |
| Atefeh Salehi-Iran(2016) | Trained husband/friend or relative/control |
| Erica Schytt-Sweden(2022) | Trained community based doula |
| Nahid Bolbol-Iran(2016) | Midwifery students |
| Breat-Belgium (1992) | Midwife |
| Breat-France (1992) | Midwife |
| Breat-Greece (1992) | Midwife |
| Torres – Chile (1999) | Companion of choice by mother |
| Dickinson-Australia (2002) | Midwife |
| Akbarzadeh-Iran (2014) | Doula/Trained researcher |
