# Supplementary material for: Impact of continuous labor companion- who is the best: A systematic review and meta-analysis of randomized controlled trials
Source: PLoS One. 2024 Jul 23;19(7):e0298852. doi: 10.1371/journal.pone.0298852 (PMC11265680; doi:10.1371/journal.pone.0298852)
Supplement: Attachment — Submitted filename: PLOS ONE Review March 25 2024.docx [file pone.0298852.s015.docx]

**Manuscript #:** PONE-D-24-02940

**Title:** Impact of continuous labour companion- who is the best: A comprehensive meta-analysis on familiarity, training, temporal association, and geographical location

**Authors:** DMCS Jayasundara; IA Jayawardane; SDS Weliange; TDKM Jayasingha; TMSSB Madugalle

**Article type:** Research Article

Thank you for the opportunity to review this important work.

I should congratulate the authors on this meticulous work, which is publishable in PLOS ONE. However, I would like to suggest a few comments to improve the scientific validity and readability of the manuscript.

**Title**

Suggest adding a ‘systematic review and meta-analysis of randomised controlled trials’ to the title. Please make sure the title is not too long.

**Abstract**

Mention the timeline of your search.

Please explain how funnel plots are used to assess the risk of bias. Funnel plots are used to assess the publication bias.

Prospective registration of the protocol indicates the transparency of the work. Could you please mention whether this work was registered prospectively? If not, explain.

CI – You need to word it out before using it as an abbreviation.

APGAR – This is not an abbreviation. Regina Apgar introduced this score. Please correct this as Apgar throughout the manuscript.

Line 40-43: Please add the summary statistics for the results. There is a difference between a lay language summary and a scientific abstract. Please have a look at a Cochrane review of the way they have presented the results.

Line 44: Add systematic review and meta-analysis. It is unclear why the authors mentioned underscoring the benefits of labour companionship. It is not clear; please rephrase.

The discussion/conclusion needs to be rephrased for clarity.

**Main text**

Line 96-97: Tokophobia and tokophobia are used interchangeably. Please use one. Please provide a definition for tocophobia.

As this topic is interesting and broad, it would be great if the authors could introduce a few subheadings to each section. The introduction can be divided into background/literature review/gap of knowledge/objectives, etc. The same applies to the other sections: methods, results, and discussion.

Line 106: was the search from inception to 04-07-2023? Please mention.

Line 177: Can you provide references for these meta-analyses? Perhaps you could discuss these with your findings in the discussion section.

Line 118: I suggest this part should go into a subheading, such as screening eligible studies. Please mention the initials of each author responsible for reviewing. Generally, conflicts between the primary and second reviewers are resolved by a third reviewer, if required.

Please add a sub-title explaining all the categories: familiar versus unfamiliar, trained versus untrained, and what temporal association is.

How did you divide the geographical territory? WHO has a categorization.

Line 130: add ‘viable’ singleton pregnancies

Line 130: early labour, more accurately, the latent phase.

Line 132: please explain what you mean by genital abnormalities here

Lines 133-134: What about quasi-experimental studies? Otherwise, you can simply mention that any other study designs, other than randomised controlled trials, including quasi-experimental trials, were excluded. Did you have limitations for the article language? Were all the articles in English?

Line 135: Suggest not mixing the study outcomes with the data extraction and quality assessment. Study outcomes should be a separate subtopic followed by another subtopic for the ‘Risk of bias and quality assessment’- RoB2 and possible biases. In the study outcomes, please mention primary and secondary outcomes clearly.

Line 144-146: Risk of bias assessment – I am not sure the authors have used the current RoB -2 tool as the domains that appear on the manuscript are different from the RoB -2. Looks like authors have used RoB -01. Please update accordingly.

**Please go to this link for more information on RoB 2:** https://sites.google.com/site/riskofbiastool/welcome/rob-2-0-tool/current-version-of-rob-2

Line 150-160: Suggest using ‘versus’ instead of ‘vs’.

Table 1: Please provide what LSCS means as a footnote. All tables/figures should be self-explanatory. Can not use RCT in the table legend. Please word it out.

Table 1: What is the order of appearance of the RCTs in Table 1? It should be either the chronological order of appearance, A-Z or any other rational categorization.

Table 1, 6^th^ column: what are these outcomes? Are these the primary outcomes reported by the trials? Or else all the outcomes? It would be more explanatory to divide primary and secondary outcomes.

Line 219: There cannot be more than one funnel plot to assess the publication bias for one meta-analysis. Could you please elaborate more?

Line 220-221: Inability to blind is a limitation; please acknowledge this in the discussion.

In Tables 2/3/4, I2 for heterogeneity appears as a decimal value. Is this a mistake? According to the Cochrane Handbook, I2 should be 0-100% as a percentage.

Line 260: cesarean section, which is abbreviated as LSCS. Please correct.

Suggest American English terms like labor, not ‘labour’ throughout the manuscript.

Table 4: 6^th^ column – please explain what you mean by the test for subgroup difference.

**Discussion:**

- The first paragraph needs to be rewritten for clarity. It should start with the research gap and objective and end with the main findings.
- Please divide the entire discussion into separate sections as sub-topics to increase the readability of the paper. Please follow the steps of a well-written systematic review publication in a reputed journal. For example: Main findings, Critical review of literature, Strengths and limitations, Conclusions, Recommendations, Future directions etc.
- Line 312: Define ICU/SCBU abbreviations

**Reference list:**

None of the references have included the journal name.
