# Supplementary material for: Impact of continuous labor companion- who is the best: A systematic review and meta-analysis of randomized controlled trials
Source: PLoS One. 2024 Jul 23;19(7):e0298852. doi: 10.1371/journal.pone.0298852 (PMC11265680; doi:10.1371/journal.pone.0298852)
Supplement: Attachment — Submitted filename: Response to Reviewers.docx [file pone.0298852.s017.docx]

**We thank the reviewers and the editorial office for the excellent comments, suggestions, and advice on improving our meta-analysis. We have accepted all comments, and the article was amended accordingly. Details of the amendments are given below.**

Comments to the Author

Reviewer #1: *Thank you for the opportunity to review the revised version of the article. I think that the discussion section is much improved and that the article is ready for publication.*

**Thank you for the encouraging comments**

Reviewer #1: *One small suggestion to potentially consider is to perhaps unravel some of the complexity relating to the issue of training and familiarity in the paragraph: 'Training of labor companions versus making trained labor companions familiar'. I thought it might be helpful to state explicitly that we aren't sure if the importance of familiarity is much more significant than the training labour companions receive and that familiar companions tend not to have received any training or whether the training being provided is somehow reducing the effectiveness of labour companions who would otherwise be more effective? And the more work needs to be done to understand this relationship?*

**We thank the reviewer for the encouraging comments and agree to add clarifications and comments. We have included a brief discussion as suggested. (Lines 428-455)**

Reviewer #2: *Thank you for addressing the reviewer's comments. The manuscript is now very much improved and almost suitable for publication in this journal.*  *However, I would like to suggest a few points. Once these have been addressed, it should be suitable for publication following the editorial review.*

- Line 310: I2 is expressed as 0.44. Please check.

**Response: We thank the reviewer for the encouraging evaluation and the suggestions for improvement.**  **We have corrected it as a percentage. (Line 311)**

- Please define all abbreviations used in all tables and figures (such as RR, CI, RCT) at the bottom

as footnotes.

**Thank you. We have defined it as suggested in the included footnotes.**

- Line 305: You may discuss this finding (length of labor) in the discussion section, probably due

to the probable rise of cesarean section rates after 2000.

**Thank you. We agree. We have included the discussion (Lines 476-480)**

- Another limitation is the methodological quality of the randomized trials before 2000. There

was a huge improvement after 2000, especially with the introduction of trial registries in 2006.

These aspects are now being discussed everywhere.

**Thank you. We have included the discussion point (Lines 476-480)**

- I noticed a high risk of bias due to blinding: suggest a sentence in the limitations section/

discussion section, as blinding is not possible due to the nature of the intervention concerned.

**Thank you. We have included a sentence acknowledging the limitation (Lines 518-521)**

- Please acknowledge the use of the older version - 'original version of the Cochrane risk of bias

tool’, rather than the currently accepted RoB-02 tool. This is an important limitation. You may also replace the word 'RoB - 01' with the name of 'original version of the Cochrane risk of bias tool’.

**Thank you. We have acknowledged the limitation (Lines 30, 154,156, 250, 520-521)**

- Finally, if the authors are happy (optional request), they can attach the Excel files/summary data

files showing the results of the data extraction used to calculate the relative risks for at least the primary outcomes. These can be added as supplementary files. This might enhance the quality of your work.

**Response: We are happy to share summary data showing the data extraction results. We have attached summary data as supplementary file 12 (S12 File).**

Congratulations on the good work.

**Thank you.**
